# Common salt aggravated pathology of testosterone-induced benign prostatic hyperplasia in adult male Wistar rat

**DOI:** 10.1186/s12894-023-01371-x

**Published:** 2023-12-11

**Authors:** Idris Idowu Bello, Akinyinka Omigbodun, Imran Morhason-Bello

**Affiliations:** 1 Department of Reproductive Health Sciences, Pan African University Life and Earth Sciences Institute (including Health and Agriculture), PAULESI, University of Ibadan, Ibadan, Nigeria; 2 Department of Animal Health Technology, Oyo State College of Agriculture and Technology, Igboora, Oyo State Nigeria; 3 Department of Obstetrics and Gynaecology, Faculty of Clinical Sciences, College of Medicine, University of Ibadan, Ibadan, Nigeria; 4 Institute for Advanced Medical Research and Training, College of Medicine, University of Ibadan, Ibadan, Nigeria

**Keywords:** Common/dietary salt, BPH, Oxidative stress, Inflammation, Apoptosis

## Abstract

**Background:**

Benign prostatic hyperplasia (BPH) is a major health concern associated with lower urinary tract symptoms and sexual dysfunction in men. Recurrent inflammation, decreased apoptotic rate and oxidative stress are some of the theories that explain the pathophysiology of BPH. Common salt, a food additive, is known to cause systemic inflammation and redox imbalance, and may serve as a potential risk factor for BPH development or progression. This study examined the effect of common salt intake on the pathology of testosterone-induced BPH.

**Methods:**

Forty male Wistar rats were randomly divided into four equal groups of 10: a control and three salt diet groups-low-salt diet (LSD), standard-salt diet (SSD) and high-salt diet (HSD). The rats were castrated, allowed to recuperate and placed on salt-free diet (control), 0.25% salt diet (LSD), 0.5% salt diet (SSD) and 1.25% salt diet (HSD) for 60 days *ad libitum.* On day 33, BPH was induced in all the rats with daily injections of testosterone propionate-Testost® (3 mg/kg body weight) for 28 days. The rats had overnight fast (12 h) on day 60 and were euthanized the following day in order to collect blood and prostate samples for biochemical, molecular and immunohistochemistry (IHC) analyses. Mean ± SD values were calculated for each group and compared for significant difference with ANOVA followed by post hoc test (Tukey HSD) at *p* < 0.05.

**Results:**

This study recorded a substantially higher level of IL-6, IL-8 and COX-2 in salt diet groups and moderate IHC staining of COX-2 in HSD group. The prostatic level of IL-17, IL-1β, PGE2, relative prostate weight and serum PSA levels were not statistically different. The concentrations of IGF-1, TGF-β were similar in all the groups but there were multiple fold increase in Bcl-2 expression in salt diet groups-LSD (13.2), SSD (9.5) and HSD (7.9) and multiple fold decrease in VEGF expression in LSD (-6.3), SSD (-5.1) and HSD (-14.1) compared to control. Activity of superoxide dismutase (SOD) and concentration of nitric oxide rose in LSD and SSD groups, and SSD and HSD groups respectively. Activities of glutathione peroxidase and catalase, and concentration of NADPH and hydrogen peroxide were not significantly different. IHC showed positive immunostaining for iNOS expression in all the groups while histopathology revealed moderate to severe prostatic hyperplasia in salt diet groups.

**Conclusions:**

These findings suggest that low, standard and high salt diets aggravated the pathology of testosterone-induced BPH in Wistar rats by promoting inflammation, oxidative stress, while suppressing apoptosis and angiogenesis.

## Background

Benign prostatic hyperplasia is characterized by excessive proliferation of stromal and epithelial cells in the transition zone of the prostate. It is associated with lower urinary tract symptoms and poor quality of life in adult men [1]. Worldwide, the global prevalence of BPH has remained 26.2% for more than two decades [2]. The economic burden of BPH and accompanying complications is estimated to be above US $180 million, US $1100, US $300-1,300 in United States of America, Ghana and Nigeria respectively [3–5].

At present, the etiology of BPH is unknown [6]. However, there are several hypotheses on the roles of androgens, inflammation, oxidative stress and diets in prostate enlargement. Reports of some studies have shown that androgens such as dihydrotestosterone and testosterone caused BPH by promoting inflammation, oxidative stress [7] and by modulating the expression of growth regulatory proteins including insulin-like growth factor (IGF) [8], vascular endothelial growth factors (VEGF) [9, 10], and apoptosis suppressor, B-cell lymphoma-2 (Bcl-2) [7]. These growth stimulatory proteins induce mitogenesis of prostate cells, resulting in hyperplasia and hypertrophy of the prostate. In spite of the strong association between androgens and BPH development, they are considered to be partly responsible for BPH pathophysiology [11].

Oxidative stress (OS) refers to an imbalance between production of free radicals and their elimination by antioxidant enzymes leading to accumulation of free radicals. Antioxidant enzymes- catalase, superoxide dismutase (SOD), glutathione peroxidase (GPx) are produced *de novo.* These enzymes usually neutralize free radicals [12]. Decreased level or activity of antioxidant enzymes results in oxidative stress, which can stimulate BPH development by causing imbalance between cell proliferation and cell death [13, 14].

Benign prostatic hyperplasia is regarded as an autoimmune disorder characterized by preponderance of inflammatory cells [15] and increased expression of pro-inflammatory mediators such as interleukin (IL)-17, IL-1 IL-6, IL-8, tumour necrotic factor-alpha (TNF-α), and cyclo-oxygenase-2 (COX-2) in prostate tissue [16]. The chronic inflammation in BPH might be due to infection, autoimmune diseases and obesity [17, 18]. However, the initial stimulus for the inflammatory process in BPH remains unknown [19]. The chronic inflammation in BPH usually upregulates the expression of growth factors [20, 21] and triggers recurrent injury and healing of prostate cells resulting into the enlargement of prostate gland [22].

Common salt (NaCl) is the main source of sodium in diets (https://www.who.int/news-room/fact-sheets/detail/salt-reduction). Generally, most people take salt that is more than the recommended maximum intake level of 5 g per day [23]. The high intake of salt is largely attributable to increasing intake of processed food by people in urban communities [17]. Consumption of large amounts of salt is associated with increased differentiation of T-lymphocytes to inflammatory T_H_17 phenotypes [24, 25] and release of pro-inflammatory cytokines such as IL-1, IL-6 and IL-17 A by immune cells [26]. Evidence from some studies showed that high levels of salt intake by rodents is associated with increased population of inflammatory cells in the kidney [27] and heart [28]. High salt levels in rodents increased cellular oxidative stress [29], and reduced activity of antioxidant enzymes in the myocardium [30, 31], hippocampus [32] and the arterial vessels [33, 34]. Interestingly, the specific level of salt that is associated with sodium toxicity and inflammation is still unknown [35, 36].

Most of the previous studies examined the pro-inflammatory and oxidative stress inducing properties of high level of salts in tissues like kidney, liver, heart and arteries. There is dearth of data on the role of salt in the development or severity of BPH. The goal of this study was to determine the effect of low-salt diet (LSD), standard-salt diet (SSD) and high-salt diet (HSD) on the pathology of testosterone-induced BPH in Wistar rats.

## Methods

### Source of animal, salt and testosterone

Forty adult male Wistar rats (16 weeks old) weighing 180-200 g were used for this study. Common salt and testosterone used for this experiment were bought at a local market and a government approved pharmacy shop in Ibadan respectively.

### Experimental site and management

This experiment was conducted at the Central Animal House, University of Ibadan. The rats were housed in eight clean and well ventilated cages covered with wire mesh in an environment with 12- hour light-dark cycle. Water and feeds were provided *ad libitum* to the animals throughout the duration of this experiment. They were allowed to acclimatize to the laboratory environment for two weeks before the commencement of the study. This study was carried out in accordance with ARRIVE guidelines.

### Sample size determination

Sample size of ten animals per group was considered for this study after consideration of attrition that could occur as a result of complications of castration and adequacy of samples for laboratory analysis. To determine adequacy of number of rats allocated per group, the resource equation method described below was used [37].

### Resource equation

E (degree of freedom of analysis of variance) = Total number of animals -Total number of groups.

 Four experimental groups with total number of forty rats were used in this study.

Therefore

$$\mathrm E=\;\left(10\;\times\;4\right)\;-\;4\dots\dots\dots\dots\mathrm E\;=\;36$$

The value of E must lie between 10 and 20 and when it is greater than 20, then adding more animals will not increase the chance of getting significant results [38]. The *E* value in the present study is greater than 20, hence the sample size is more than enough.

### Study design

Forty adult male Wistar rats were assigned into four equal groups of 10 using simple randomization: control group and three (3) salt diet groups- low-salt diet (LSD), standard-salt diet (SSD) and high-salt diet (HSD).

### Experimental phases

The experiment was divided into preparatory and exposure phases.

#### Preparatory phase

In the preparatory phase, the animals were castrated to prevent the possible influence of endogenously produced testosterone on BPH induction. The rats were anaesthetized before castration by using intra-peritoneal injection of anaesthetic agents, xylazine 0.2 ml/kg and ketamine-25 mg/kg. The castration was performed by making an incision on the scrotal septum. The testicles and epididymal fat were removed through the scrota sac using the protocol of Van Coppenolle et al. [39]. Thereafter, the blood vessels and spermatic cord were ligated with absorbable sutures and the redundant tissues were excised. Penicillin-streptomycin (Pen-Strep) antibiotic was administered to the rats via drinking water (0.5 g of Pen-Strep/1 litre of water) to prevent infection that could occur following the castration procedure. The rats were allowed to recuperate for 2 weeks before the beginning of the exposure phase. All the rats were placed on commercial feed (Top Feed ®) during the preparatory phase.

#### Exposure phase

The exposure phase involved feeding the rats with diets containing varying quantities of salt for 60 days and induction of BPH for 28 days.

##### Duration of exposure phase

The exposure phase commenced immediately after expiration of timeline left for recovery from castration and spanned for 60 days.

##### Feed formulation and placement of rats on common salt-enriched diet

Salt-free feed was compounded at a commercial feed-mill in Ibadan as described in a previous study [40]. The feed was divided into four equal portions. After, different amount of salts were added: 2.5 g of salt/1 kg feed (LSD- 0.25% salt in the diet), 5 g of salt/ 1 kg feed (SSD- 0.5% salt in the diet) and 12.5 g of salt/1 kg feed (HSD − 1.25% salt in the diet). The fourth group is the control that was placed on salt-free diet. The animals were fed their respective diets for 60 days.

##### Commencement of BPH induction and duration

Day 33 of the exposure phase, BPH was induced by subcutaneous administration of testosterone propionate (Testost®) (3 mg/kg) for 28 days as previously described [41].

### Animal sacrifice and sample collection

The rats were fasted overnight at the end of the exposure phase and sacrificed the next morning via intra-peritoneal injection of 0.3ml of thiopentone/0.2 kg body weight. The rats were thereafter cut open with the use of scissors and scapel blade from the abdominal cavity to the thorax and inguinal regions to access the heart and prostate glands respectively. Blood samples for PSA analysis were collected via cardiopuncture into covered plain bottles. The blood samples were centrifuged for 15 min at 3000 rpm to obtain serum for PSA analysis. Prostate glands were excised, cleared of adherent tissue and weighed using a sensitive weighing scale. Thereafter, prostate lobes that were harvested from five rats in each group were put in separate organ bottles and submerged with phosphate buffer solution (50 mM: pH 7.4). They were homogenized by spinning for fifteen minutes at 10,000* g*. The homogenized prostate tissues were cold centrifuged at 10,000 rpm for 15 min to collect supernatant for biochemical analysis. One of the two prostate lobes harvested from the remaining five rats in each group were preserved in separate organ bottle containing 10% buffered formalin for histopathology and IHC analysis of inducible nitric oxide synthase (iNOS) and COX-2 expression. The second prostate lobes collected for qPCR analysis of VEGF and Bcl-2 were put in organ bottles and submerged with 0.5 ml RNAlater ^TM^ (Sigma ® Life Science).

### Determination of relative prostate weight

The relative prostate weight of each animal was determined as previously described [35]:

$$\mathrm{Relative}\;\mathrm{organ}\;\mathrm{weight}=\frac{\mathrm{Absolute}\;\mathrm{weight}\;\mathrm{of}\;\mathrm{organ}\;\left(\mathrm g\right)}{\mathrm{Body}\;\mathrm{weight}\;\mathrm{of}\;\mathrm{rat}\;\mathrm{on}\;\mathrm{sacrifice}\;\left(\mathrm g\right)}\times100$$

### Laboratory analysis

#### Histology and immunohistochemistry analysis

The prostate tissue sections were dehydrated using ethanol (95%), and later embedded in paraffin. Thereafter the tissues were cleared with xylene. After, 5 μm sections of prostate tissues were cut from paraffin embedded gland and stained with hematoxylin and eosin. The tissue histology was observed under light microscope for histopathological lesions. Tissue sections of 5 μm thickness were mounted on slides and prepared for iNOS and COX-2 IHC staining as previously described using primary antibodies, iNOS Rabbit pAb (FNab10777) and COX-2 Rabbit pAb (FNab10407), and secondary antibody, Goat Anti-Rabbit IgG (FNSA-DO41).

#### Analysis of serum PSA

Serum concentrations of PSA were quantified by solid phase enzyme linked immunosorbent assay (ELISA) following manufacturer’s procedure (Rat ELISA Kit, Calbiotech, Inc, Spring Valley, CA, USA) [42].

#### Analysis of inflammatory markers

The concentrations of IL-17, IL-1β, IL-6, IL-8, TNF-α and PGE2 were quantified using the following ELISA assays (Elabscience®)- E-EL-R0566, E-EL-R0012, E-EL-R0015, E-EL-RB1142, E-EL-R2856 and E-EL-0034, respectively in accordance with manufacturer’s procedure. The activity of COX-2 was determined as described in a previous study [43]. This involved measuring the peroxidase activity of cyclooxygenase by monitoring the appearance of blue oxidized N,N,N^’^,N^’^-tetramethyl-pphenylenediamine which reflects the rate of conversion of arachidonic acid to PGH2.

#### Analysis of oxidative stress marker

The activity of GPx was determined using assay kit (Fortress diagnostic kit, Ltd., Atrim, UK BXC0551) following manufacturer’s procedure. Catalase activity was determined using assay kit (Elabscience, USA, E-BC-K031-S) in accordance with manufacturer’s instruction. The principle of assay is based on the measurement of a complex formed from reaction of H_2_O_2_ with ammonium molybdate at 405 nm. The activity of SOD was determined using protocol described by Marklund and Marklund [44]. The principle of this method is based on the competition between the pyrogallol autoxidation by O_2_^•^¯ and the dismutation of this radical by SOD. Prostate MDA levels were measured using TBARS method as described by a previous study [45]. Concentrations of H_2_O_2_ in prostate tissues homogenate were determined by measuring absorbance value of yellow complex formed from reaction of H_2_O_2_ with ammonium molybdate at 405 nm. The quantity of nitrite in prostate tissue was determined using Griess’ reagent as described by Palmer et al. [46].

#### Analysis of growth regulatory protein

The concentrations of TGF-β and IGF-1 were quantified by ELISA using ELISA Kit (Elabscience®)-E-EL-0162 and E-EL-R3001 respectively in accordance with manufacturer’s procedure. The expressions of VEGF and Bcl-2 were quantified via extraction of Total RNA from prostate tissue, followed by reverse transcription of extracted RNA using FIREScript® RT cDNA Synthesis KIT (Thistle Scientific, UK), preparation of reaction mixture containing forward and reverse primers- BCL-2 and VEGF (Table 1) and running of real-time PCR for target genes and house-keeping gene- glyceraldehyde-3-phosphate dehydrogenase (GAPDH) on MyiQ ^TM^ Single Color Real-Time PCR Detection System (Bio-Rad, Conquer Scientific, United State). Lastly, ΔCt (threshold cycle) was determined and thereafter, relative fold change of VEGF and Bcl-2 changes in target gene expression were calculated using the formula 2^−ΔCt^.

**Table 1:** Primer Sequence of Bcl-2 and VEGF

Primer	Forward	Reverse
Bcl-2	5′-GCAGCTTCTTTCCCCGGAAGGA	5′-AGGTGCAGCTGACTGGACATCT
VEGF	5′-CTCCACCATGCCAAGTGGTC	5′-AATAGCTGCGCTGGTAGACG

### Data analysis

The data were entered analyzed with Statistical Package for the Social Sciences (SPSS) version 23.0 for windows. The normality of the data in each group was tested with Shapiro Wilk and normality assumption was considered to be violated at *P* < 0.05. The means of control and salt diet groups were tested for significant difference with one-way ANOVA followed by post hoc analysis using Tukey HSD. A value of *P* < 0.05 was considered statistically significant. The mean ± SD was presented for each group.

## Results

### Inflammatory markers

The concentration of IL-17, IL-1β, PGE2 in salt diet groups were not significantly different from value recorded in the control group. Although HSD group had the highest IL-8 and IL-6 concentrations, IL-8 and IL-6 levels as well as COX-2 activity were significantly higher in all salt diet groups relative to the control group. IL-17, IL-1β, PGE2, IL-8, IL-6 concentrations, and COX-2 activity in salt diet groups were not significantly different when compared with each other (Table 2).

**Table 2:** Concentration of inflammatory markers in prostate tissue

Parameters	Control	LSD Group	SSD Group	HSD Group	*P* value
IL-17 (pg/ml)	168.09 ± 54.27	130.72 ± 32.09	140.25 ± 19.33	112.43 ± 16.53	0.113
IL-8 (pg/ml)	349.85 ± 65.19	1632.08 ± 497.99^*^	1587.31 ± 407.04^*^	2286.86 ± 337.76^*^	0.001
IL-1β (pg/ml)	41.36 ± 67.60	16.49 ± 13.89	40.46 ± 38.15	37.267 ± 32.98	0.771
IL-6 (pg/ml)	340.21 ± 51.92	2810.15 ± 966.18^*^	1038.55 ± 232.26^*^	2910.60 ± 629.31^*^	0.01
COX-2 (U/L)	3.44 ± 2.07	7.44 ± 0.85^*^	9.61 ± 1.96^*^	7.99 ± 3.28^*^	0.003
PGE2 (pg/ml)	17.60 ± 4.83	12.55 ± 6.84	17.53 ± 10.50	13.53 ± 6.61	0.610

*Indicates significant difference when compared with the control (*p* < 0.05)

### Oxidative stress markers

NADPH and H_2_O_2_ levels were greater in salt diet groups but not statistically significant compared with the control. Also, MDA concentration as well as catalase and GPx activities in salt diet groups were not significantly different from values recorded in the control group. Generally, activity of SOD and NO increased in salt diet groups but only significant in LSD and HSD, and SSD and HSD groups respectively relative to the control. When salt diet groups were compared with each other, concentrations of NADPH, MDA, H_2_O_2_ and nitric oxide, and activities of catalase, GPx and SOD were not significantly different (Table 3).

**Table 3:** Oxidative stress markers in prostate tissue

Parameters	Control	LSD Group	SSD Group	HSD Group	*P* value
NADPH (ng/ml)	24.96 ± 1.84	27.33 ± 2.47	28.25 ± 3.28	28.52 ± 3.27	0.208
MDA (uM)	0.87 ± 0.55	0.88 ± 0.24	0.62 ± 0.45	0.50 ± 0.43	0.230
GPx (U/L)	36.91 ± 13.86	25.43 ± 19.12	22.51 ± 10.25	29.26 ± 19.77	0.214
Catalase (U/ml)	9.43 ± 3.25	6.63 ± 2.37	9.32 ± 0.99	9.32 ± 4.37	0.407
SOD (U/ml)	0.38 ± 0.07	0.62 ± 0.13^*^	0.66 ± 0.05^*^	0.59 ± 0.19	0.035
H_2_O_2_ (mM)	2.31 ± 0.16	2.42 ± 0.40	2.59 ± 0.28	2.50 ± 0.34	0.575
NO (uM)	6.49 ± 0.26	7.08 ± 1.49	7.11 ± 0.16^*^	7.95 ± 1.01^*^	0.025

*Indicates significant difference when compared with the control (*p* < 0.05)

#### 3.2. Relative prostate weight, PSA and growth factors

**Fig. 1:**
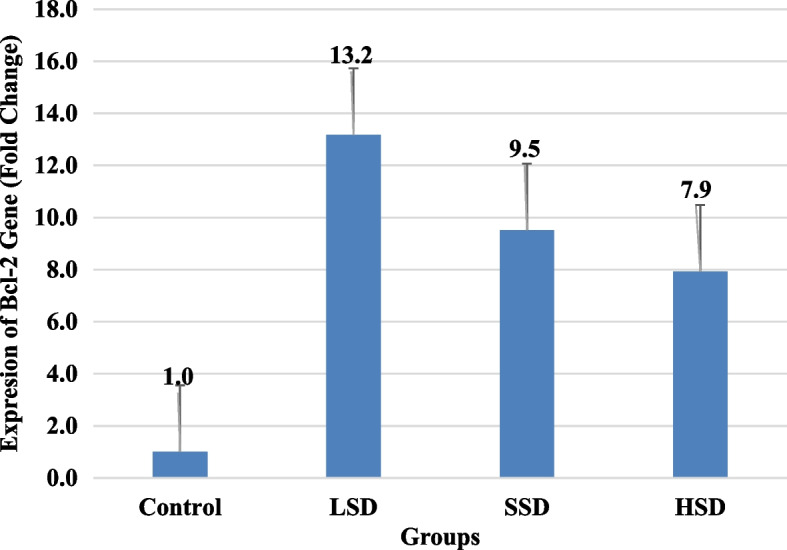
Expression of Bcl-2 in castrated male Wistar rats placed on salt diets

**Fig. 2:**
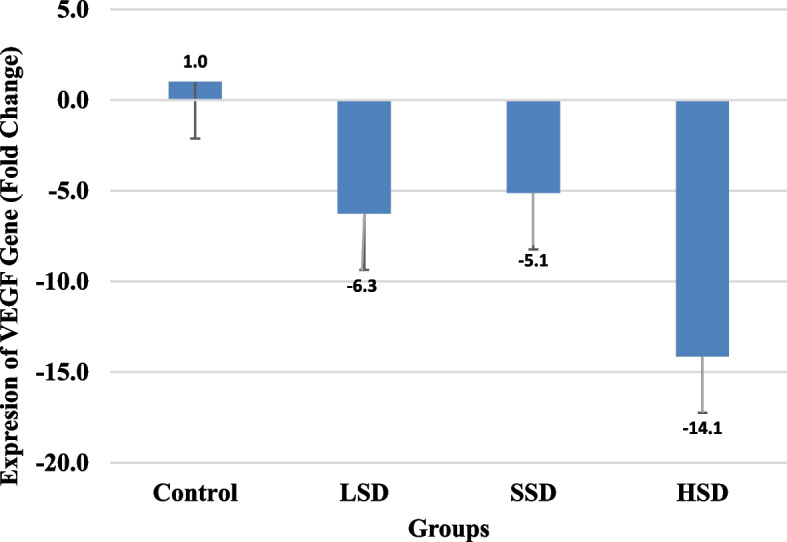
Expression of VEGF in castrated male Wistar rats placed on salt diets

Relative prostate weight, blood PSA and prostatic IGF-1, TGF-β, TNF-α levels of groups on salt diet were not significantly different from that of control group. Also, the value of these biomarkers in salt diet groups showed no significant difference when compared with each other (Table 4). Though, Bcl-2 gene expression was generally higher in all groups placed on salt diets compared to the control. However, there was an inverse relationship between Bcl-2 expression and salt concentration with the highest (13.2) fold increase in expression among those with LSD (Fig. 1). Regarding VEGF gene expression, the degree of expression was lowest (-15.1) in those with high salt diet relative to other groups placed on lower or no salt diets (Fig. 2).

**Table 4:** Relative prostate weight, PSA and growth regulatory proteins level

Parameters	Control	LSD Group	SSD Group	HSD Group	*P* value
Relative Prostate Weight (%)	0.21 ± 0.06	0.24 ± 0.04	0.21 ± 0.04	0.24 ± 0.03	0.146
PSA (ng/ml)	0.23 ± 0.02	0.26 ± 0.04	0.22 ± 0.04	0.23 ± 0.03	0.085
IGF-1 (ng/ml)	0.92 ± 0.15	1.32 ± 0.56	0.99 ± 0.15	1.104 ± 0.47	0.382
TGF-β (pg/ml)	17.57 ± 6.32	27.07 ± 22.67	14.61 ± 15.23	19.36 ± 15.40	0.653
TNF-α (pg/ml)	0.15 ± 0.03	0.17 ± 0.07	0.16 ± 0.05	0.33 ± 0.22	0.102

### Histopathology of prostate of male Wistar rats in control and salt diet groups

**Fig. 3:**
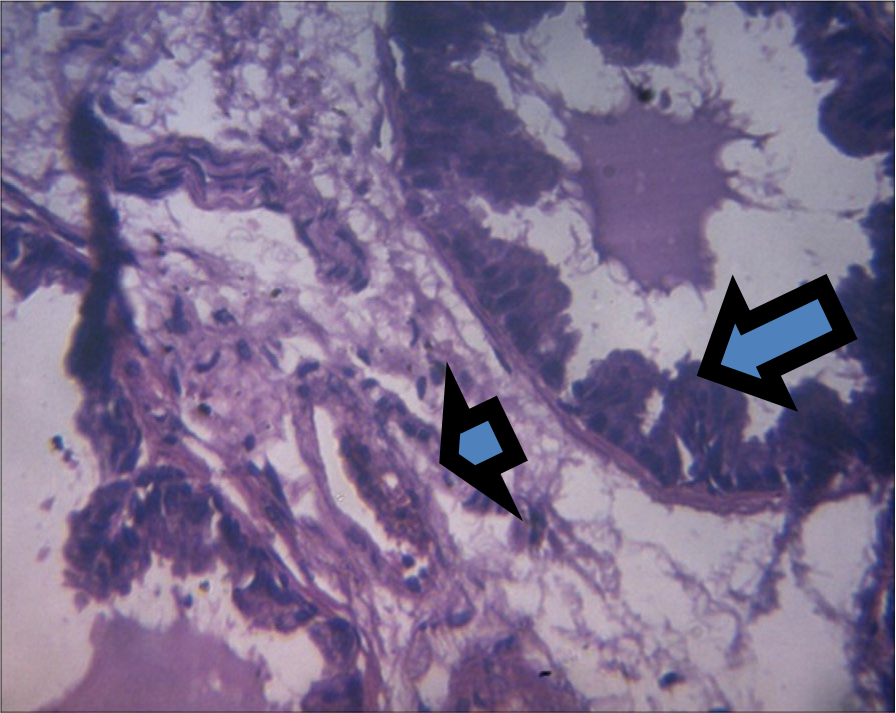
Section shows mild epithelial (arrow) and stromal hyperplasia (arrowhead) x 100

**Fig. 4:**
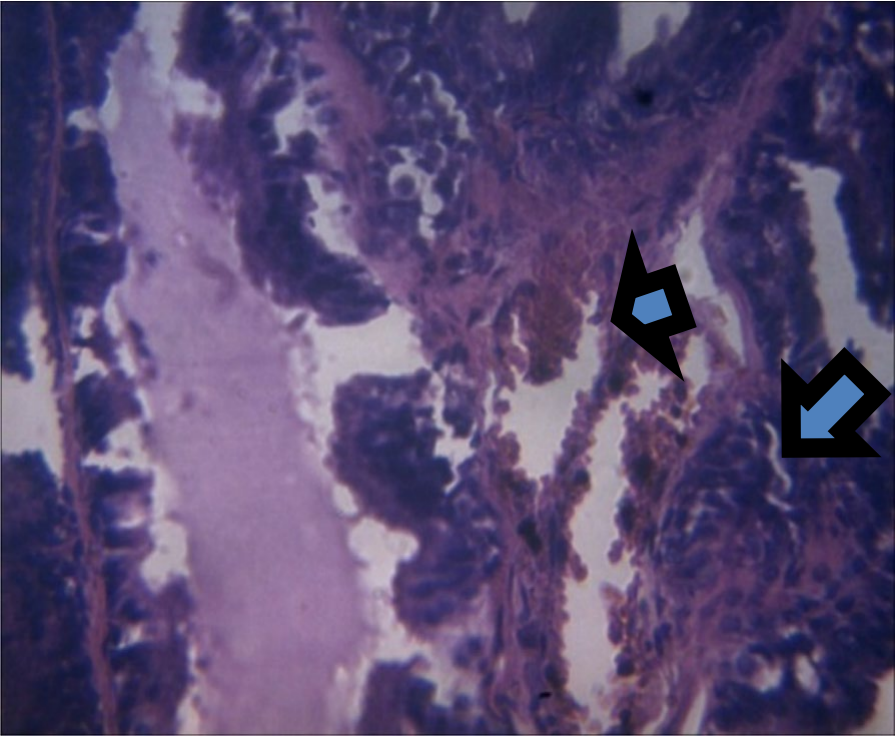
Section shows mild epithelial hyperplasia (arrow) and moderate stromal congestion (arrowhead) x 100

**Fig. 5:**
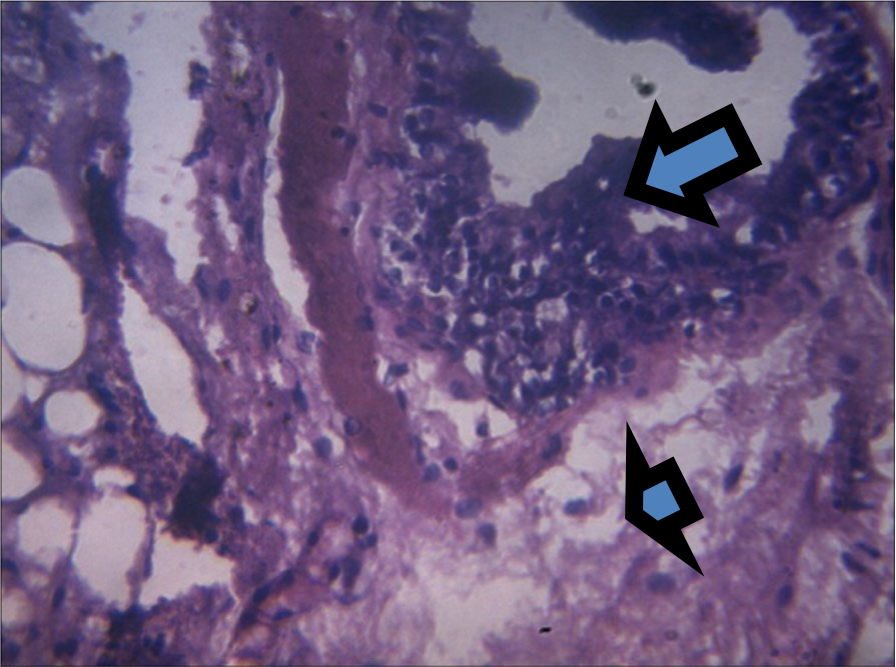
Section shows moderate epithelial (arrow) and stromal hyperplasia (arrowhead) x 100

**Fig. 6:**
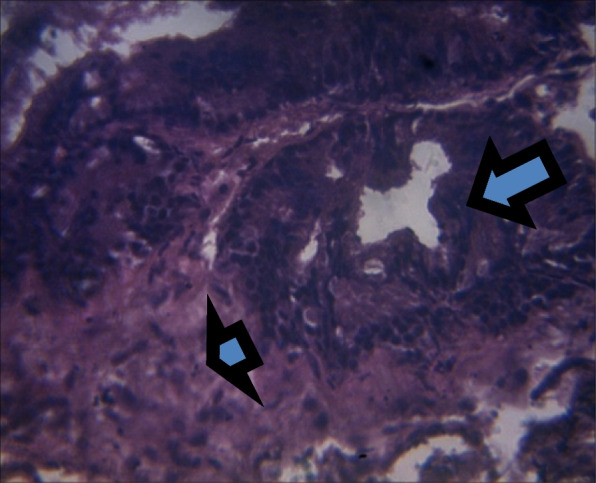
Section shows severe epithelial (arrow) and stromal hyperplasia (arrowhead) x 100

 On histology, there was a mild epithelial hyperplasia in control group (Fig. 3) and LSD group (Fig. 4), and moderate and severe epithelial hyperplasia in SSD (Fig. 5) and HSD (Fig. 6) respectively. In addition, a mild and severe stromal hyperplasia was observed in the control and HSD groups respectively but both LSD and SSD groups had moderate stromal hyperplasia.

### Immunohistochemistry of COX-2 and iNOS in prostate gland

IHC staining of prostate tissue revealed weak expression of COX-2 in the control (Fig. 7), LSD (Fig. 8), SSD (Fig. 9), and moderate expression in HSD group (Fig. 10). Meanwhile, there was weak expression of iNOS in prostatic tissues of rats in the control (Fig. 11), LSD (Fig. 12), SSD (Fig. 13) and HSD (Fig. 14).

**Fig. 7:**
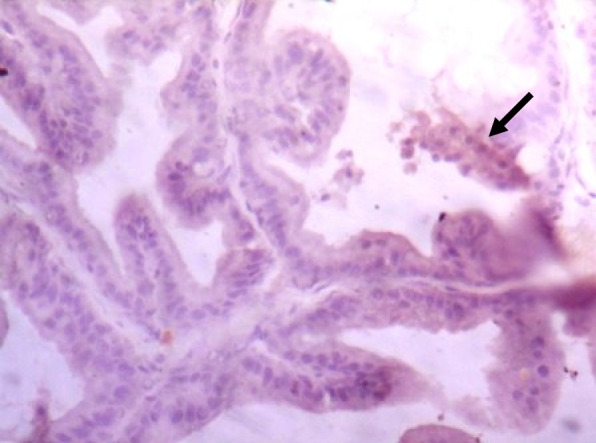
Section shows weak expression of COX-2 inthe prostate epithelium (black arrow) x 400

**Fig. 8:**
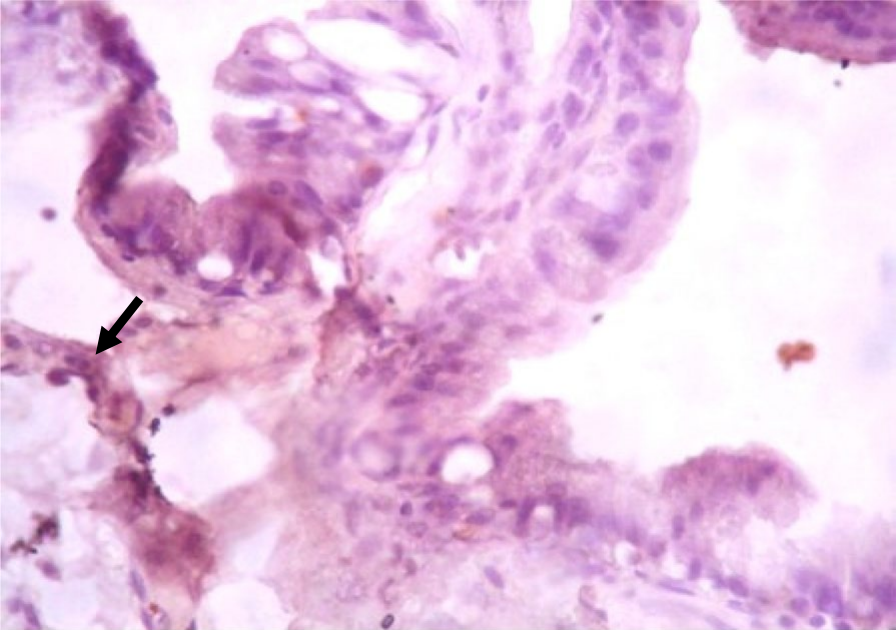
Section shows weak expression of COX-2 in the prostate epithelium (black arrow) x 400

**Fig. 9:**
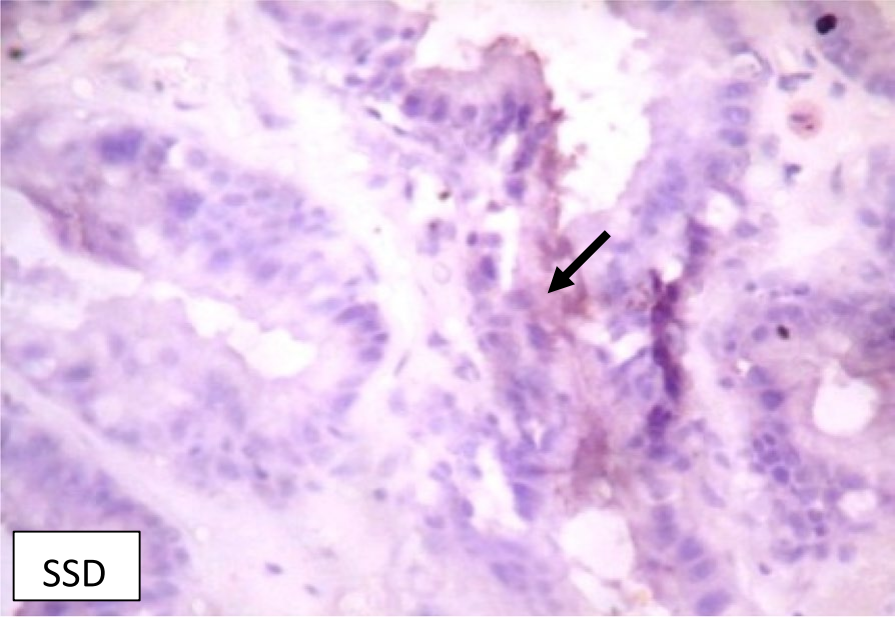
Section shows weak expression of COX-2 in prostate epithelium (black arrow) x 400

**Fig. 10:**
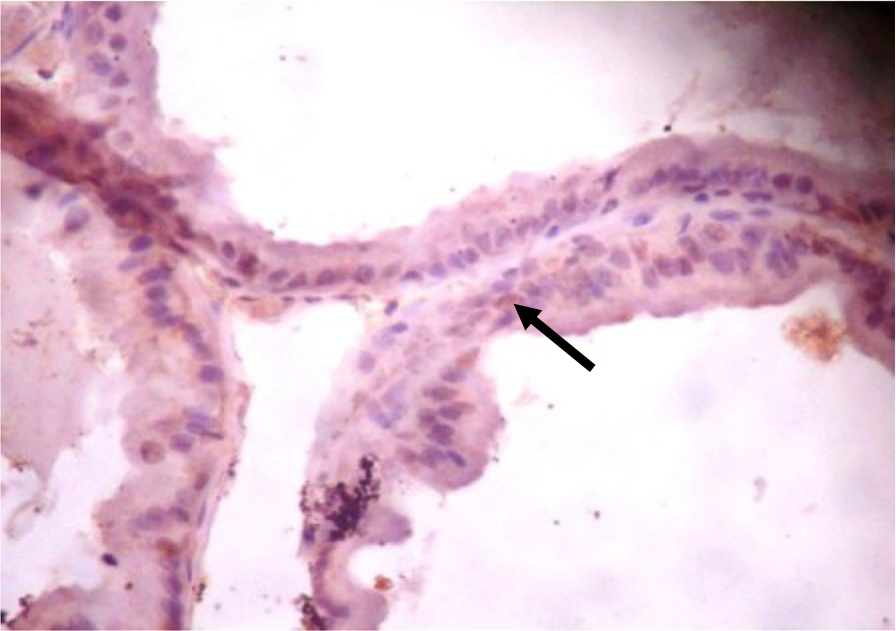
Section shows moderate expression of COX-2 in the prostate epithelium (black arrow) x 400

**Fig. 11:**
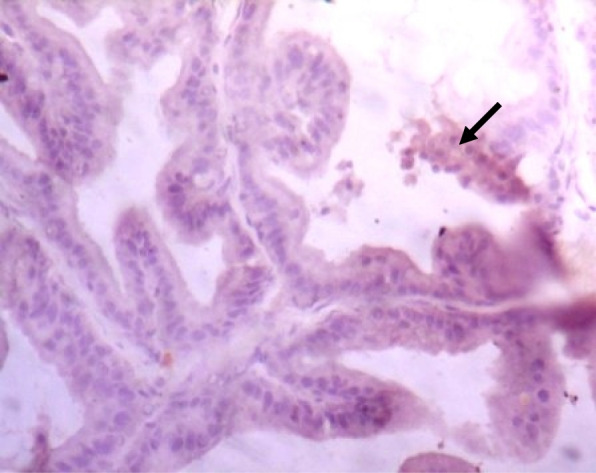
Section shows weak expression of iNOS in the prostate epithelium (black arrow) x 400

**Fig. 12:**
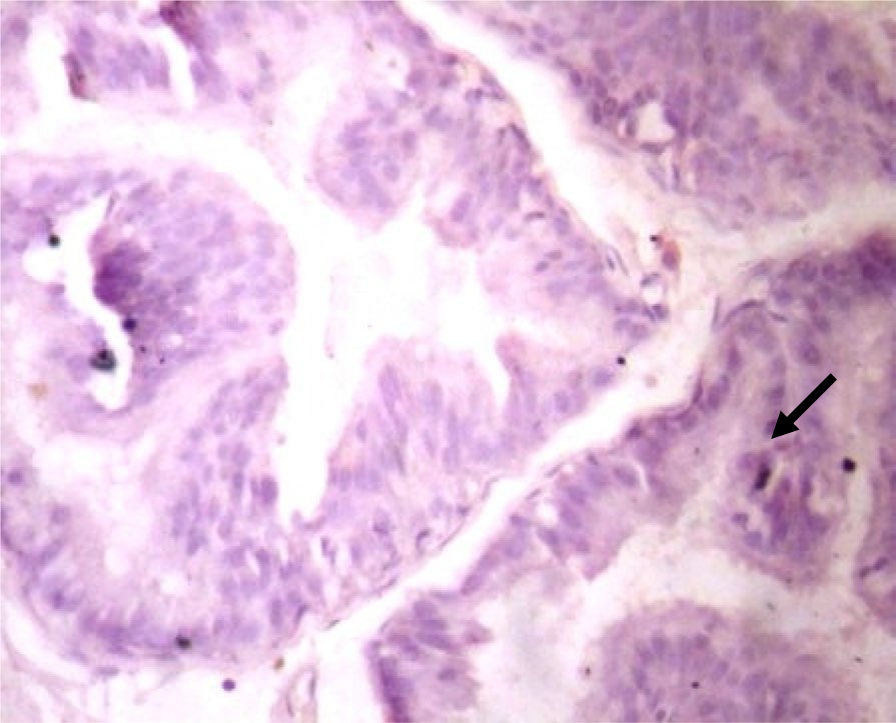
Section shows weak expression of iNOS in the prostate epithelium (black arrow) x 400

**Fig. 13:**
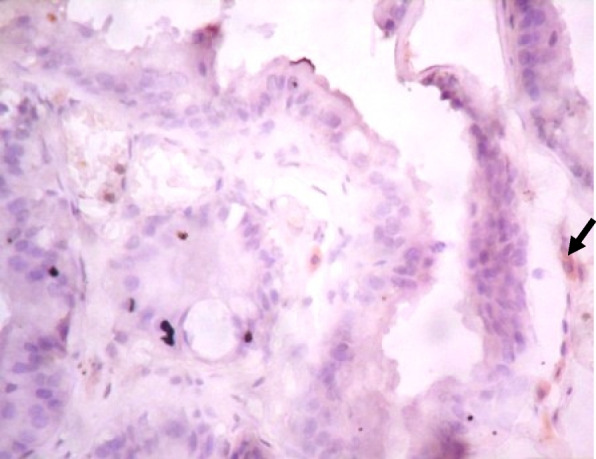
Section shows weak expression of iNOS in the prostate epithelium (black arrow) x 400

**Fig. 14:**
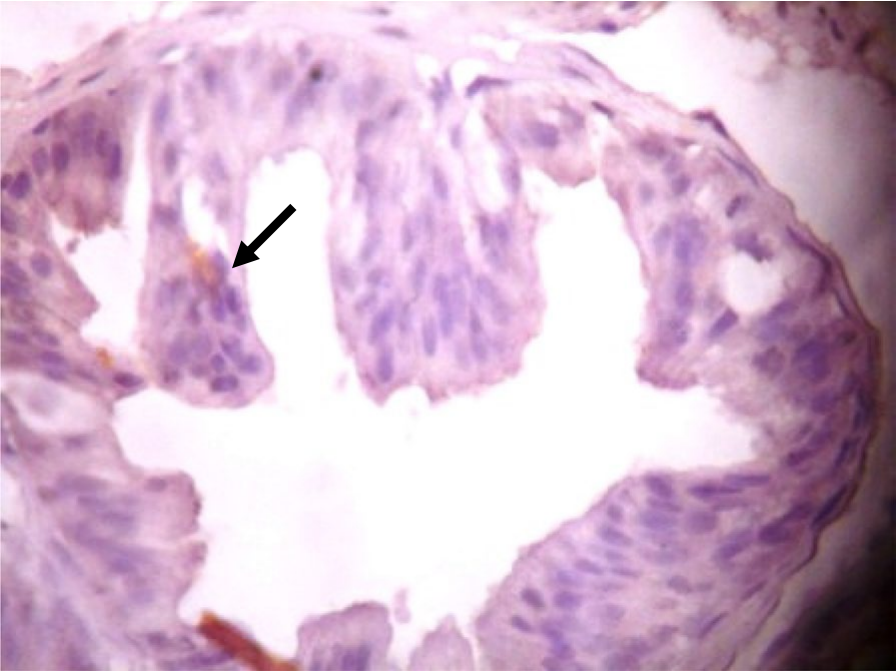
Section shows weak expression of iNOS in the prostate epithelium (black arrow) x 400

## Discussion

These findings suggest a biological relationship between salt intake and BPH development. Dietary salt worsened the pathology of testosterone-induced BPH but not in a dose-dependent pattern. The study showed that enhanced inflammatory pathway could have majorly accounted for the promotion of testosterone induced BPH following salt intake. In the current study, inflammatory pathway principally mediated the role of salt in aggravation of testosterone-induced BPH. Chronic inflammation in BPH is characterized by increased expression of pro-inflammatory mediators including of COX-2, IL-6 and IL-8 in BPH tissues. Overexpression of IL-8 and IL- 6 are associated with increased production of growth stimulating factors, FGF and IGF-1 and thereby induce prostate hyperplasia [20, 47]. Some studies have demonstrated positive correlation between serum IL-6 and IL-8 level and the occurrence of acute urinary retention in BPH patients [48, 49]. In this study, activity of COX-2 and concentration of IL-6 and IL-8 in the prostate tissues were higher in salt diet groups, indicating pro-inflammatory activity of dietary salt and its potential to aggravate BPH pathology. Given the immunocompetent nature of the prostate, this observation could be as a result of substantially higher activity or population of immune cells in the prostatic tissue of salt diet groups, leading to enhanced production of cytokines. The report linking salt to activation and function of various types of immune cells support our hypothesis [50]. In line with findings of this present study, findings of past studies showed both high and low salt diets displayed pro-inflammatory activities [51, 52]. In some other studies, high and low salt diets were also found to enhance COX-2 expression [53, 54] *and* IL-6 concentration [52, 55].

B-cell lymphoma-2 gene encodes Bcl-2 proteins, an apoptosis factor. The expression of Bcl-2 has been reported to increase following upregulation in the expression or activity of COX-2 [10, 56]. Enhanced expression Bcl-2 gene is involved in the deregulation of intrinsic apoptosis, a key factor in the initiation and progression of BPH [57, 58]. In the present study, salt-enriched diets amplified prostatic expression of Bcl-2 gene and this could be associated to elevated COX-2 activity recorded in the groups. Our finding is similar to previous report which showed an association between excessive salt intake and increased Bcl-2 signaling pathway in kidney [59].

VEGF gene codes for VEGF protein, a key mediator of hypoxia-induced-angiogenesis. A report shows that the inhibition and depletion of VEGF gene promotes tumour aggressiveness or malignancy [60]. According to Honda et al. [61], the expression of VEGF gene is stimulated by activated hypoxia inducible factor-1α (HIF-1α). Meanwhile, report has shown that HIF-1α expression decreased during severe hypoxia [62], a condition that has been reported to be induced by chronic inflammation. In the present study, prostate tissues of groups on salt-enriched diets had low VEGF expression which could be due to severe hypoxia associated with enhanced prostatic inflammation recorded in the groups. There are reports that LSD and HSD increased and reduced renal expression of VEGF respectively relative to normal salt group, suggesting inverse relationship between amount of salt intake and VEGF expression [63, 64]. These reports are in tandem with our observation that HSD group is associated with the lowest VEGF expression.

Evidence abounds that acute oxidative stress is associated with activation of nuclear factor erythroid 2 (NFE2)-related factor 2 (Nrf2) which in turn up-regulate expression of antioxidant enzymes including SOD, catalase and GPx [65, 66]. Superoxide dismutase, catalase and GPx are first line antioxidant enzymes. While SOD converts superoxide anions radical to oxygen and H_2_O_2_, catalase and GPx activity catalyze the breakdown of H_2_O_2_ and prevent H_2_O_2_ accumulation. Meanwhile, numerous studies have shown that H_2_O_2_ induced oxidative stress directly or indirectly by transforming to more reactive and toxic free radical such as ^∗^OH and HCIO through Fenton reaction [67]. Our study showed that LSD and HSD groups had up-regulated SOD activity while GPx and catalase activities in the prostate tissue remain unaltered, suggesting mild/acute oxidative stress following cellular damage incited by salt. This study also recorded a higher concentration of H_2_O_2_, though not significant, in groups on salt diet suggesting the presence of oxidative stress in prostatic tissues. A study reported similar finding of an increased SOD level in salt-treated cells [68].

Inducible nitric oxide synthase expression is induced by stimuli such as cytokines and hypoxia. Also, iNOS expression in the prostate is associated with prostatic inflammation [14]. iNOS expression is more common in BPH tissues relative to normal prostate [14]. Following induction, iNOS catalyzes the production of NO in large quantity. Excessive production of NO causes oxidative damage and damage to lipid membrane [66]. We observed positive immunostaining for iNOS in both prostatic tissues of control and salt diet groups, indicating presence of prostate hyperplasia in all the groups. However, the intra-prostatic concentrations of NO increased significantly in SSD and HSD groups, plausibly due to elevated inflammatory biomarkers in these groups. In corroboration of findings in this study, salt loading was found to enhance production of NO in vascular tissue and testis [14, 69].

The presence of prostatic hyperplasia in both control and salt diet groups on histology suggests that the induction of BPH was successfully achieved. However, moderate to severe prostatic hyperplasia was observed in SSD and HSD groups relative to other groups. The duration of exposure of rats to salt diet could account for the statistically insignificant increase in relative prostate weight and serum PSA despite histological evidence of severe hyperplasia and amplified inflammatory response in salt diet groups.

To the best of our knowledge, this study provides the first evidence of the association between different doses of salt intake and risk of BPH in rats. However, there are some potential limitations to our findings. First of all, our study did not evaluate lower urinary symptoms which could have provided more insight into the severity of BPH in the control and salt diet groups. Secondly, we assumed that the sodium content of the salt used are similar but this assumption may not be entirely true.

## Conclusion

This study demonstrated that rat diets containing low, standard and high quantity of dietary salt promoted inflammation/oxidative stress, and also inhibited apoptosis and angiogenesis in prostatic tissues. It is therefore concluded that dietary salt intake aggravated the pathology of testosterone-induced BPH rat. These observations suggest that dietary salt intake is a contributory risk factor for BPH. We recommend more animal studies to examine the causal role of salt intake in the initiation or induction of BPH.

## Data Availability

All the raw datasets generated and/or analysed during the current study available from corresponding author on reasonable request.
